# Outcome and safety 90 days after combined airway valve treatment of the right upper and middle lobes in patients with severe pulmonary emphysema

**DOI:** 10.1186/s12931-024-03069-6

**Published:** 2025-01-06

**Authors:** A. Susanne Dittrich, Cosimo Carlo De Pace, Judith Maria Brock, Franziska Trudzinski, Claus Peter Heussel, Ralf Eberhardt, Felix J. F. Herth, Konstantina Kontogianni

**Affiliations:** 1 Department of Pneumology and Critical Care Medicine, Thoraxklinik at the University Hospital Heidelberg, Heidelberg, Germany; 2 Translational Lung Research Center Heidelberg (TLRC), German Center for Lung Research (DZL), Heidelberg, Germany; 3 Department of Medical and Surgical Sciences, University of Foggia, Foggia, Italy; 4 Respiratory Diseases and Respiratory Rehabilitation, Teresa Masselli Mascia Hospital, Via 2 Giugno, San Severo, Italy; 5 Department of Diagnostic and Interventional Radiology, Thoraxklinik at the University Hospital Heidelberg, Heidelberg, Germany; 6 Department of Diagnostic and Interventional Radiology, University Hospital Heidelberg, Heidelberg, Germany; 7 Pneumology & Critical Care Medicine, Asklepios Klinik Barmbek, Hamburg, Germany

**Keywords:** Emphysema, Chronic obstructive lung diseases, Lung volume reduction, Endobronchial valve, Intrabronchial valve, Spiration System, Zephyr, Middle lobe, Right upper lobe, Fissure

## Abstract

**Background:**

In COPD patients with severe right-sided emphysema, complete major and incomplete minor fissure, implantation of one-way valves in both the right upper (RUL) and middle lobes (ML) is a possible approach for endoscopic lung volume reduction. The aim of this retrospective analysis was to evaluate the response to therapy and the complication rate at 90 days (90d-FU) after combined RUL-ML valve implantation.

**Methods:**

This retrospective, monocentric study included all patients from the Thoraxklinik Heidelberg who underwent RUL-ML valve treatment between 2012 and 2023 with available follow-up data. Quantitative chest imaging, lung function, 6-minute walking distance (6-MWD), complications and indications for re-bronchoscopies until 90d-FU were analysed.

**Results:**

28 patients underwent combined RUL-ML valve treatment, predominantly sequentially (92.86%, *n* = 26/28). Neither lung function nor 6MWD improved significantly in the overall cohort. However, in the subgroup with heterogeneous emphysema (71.4%, *n* = 20/28), FEV1 (Δ = 116.00 mL ± 195.77 mL, *p* < 0.05) and 6-MWD (Δ = 50.23 ± 69.10 m, *p* < 0.05) increased significantly at 90d-FU. Consistent with this, the baseline difference in emphysema volume between the RUL + ML and the right lower lobe correlated significantly with the increase in FEV1 at 90d-FU (*R* = 0.74, *p* < 0.001). Pneumothorax occurred in 5 cases in 4 patients (14.3%) following ML treatment. Severe pneumonia and/or COPD exacerbations occurred in 32.1% (9/28) of patients.

**Conclusions:**

Although only studied in a small cohort, our data suggest that combined RUL and ML valve implantation appears to be a promising interventional treatment strategy in patients with severe heterogenous RUL and ML emphysema.

## Introduction

Chronic obstructive pulmonary disease (COPD) is one of the a major health threats in industrialized countries and associated with significant morbidity and mortality [1]. End-stage COPD can manifest in massive pulmonary emphysema and hyperinflation, leading to respiratory exhaustion at the slightest exertion [2, 3]. Currently, endoscopic lung volume reduction (ELVR) with unidirectional airway valves is the most effective minimal-invasive strategy to treat the symptomatic burden of affected COPD patients [4]. Two airway valve systems are currently available: Zephyr valves (Pulmonx International Sarl, Neuchatel, Switzerland) and Spiration Valve System (Spiration System Inc./Olympus, Tokyo, Japan) that have been shown to be safe and effective in various randomised controlled trials [5–10]. Usually, Zephyr or Spiration valves are used to treat one single target lobe [11]. However, in presence of collateral ventilation the valve mechanism technically cannot lead to a significant reduction of lung volume or to atelectasis formation [12]. To assess whether the preferred target lobe is a treatable anatomical unit, fissure integrity is evaluated by high-resolution computed tomography (CT) and, if necessary, functional measurement using the Chartis^®^ system [12]. In the certain case of an incomplete minor, but a complete major fissure, the right upper lobe (RUL) and the middle lobe (ML) can be considered as one functional unit that is isolated from the right lower lobe (RLL) [13]. If both the RUL and the ML are significantly destructed and functionally compromised, the combined valve implantation in the RUL and in the ML evolved as a possible treatment strategy. To our knowledge, data on this specific ELVR approach are still limited. Therefore, in this retrospective analysis we aimed to collect real-life data of an experienced high-volume ELVR centre and to investigate the effectiveness and safety of combined simultaneous and /or sequential RUL-ML valve implantation.

## Materials and methods

### Study design

In this retrospective, population-based, single-center study, we collected clinical data from all COPD patients who underwent sequential (two bronchoscopies for RUL and ML each) or simultaneous valve treatment of the RUL and ML (one bronchoscopy) as well as follow-up visits at Thoraxklinik Heidelberg from December 2012 to September 2023. This study was approved by the ethics committee of the University of Heidelberg. Safety and clinical benefit at 90 days (90d-FU) after completion of RUL-ML treatment was investigated as primary endpoint. To minimise the confounding effect of the progressive lung disease on outcome analysis, lung function, patient dyspnea perception and exercise capacity were only analysed if the time between RUL and ML treatment was < 1 year. Safety analysis was performed for the whole cohort until study exit. Patients were considered to be at study end when at least one valve was permanently explanted without reimplantation or in case of permanent loss of follow-up. During 90d-FU, the clinician routinely decides whether to perform a bronchoscopic valve control based on the clinical and lung functional status of the patient. If the decision to re-bronchoscopy was made up to this point, we also recorded the outcome of these procedures (valve replacement, interval valve reimplantation or permanent valve explantation).

### Outcome parameters

Demographics, lung function, six-minute walk distance (6-MWD) and modified medical research council (mMRC) questionnaires were collected at pre-treatment baseline (BL), at 30 days after treatment of the RUL (30d-FU RUL), at 30 days after the bronchoscopy occluding the ML (30d-FU RUL + ML) and at 90 days post final procedure (90d-FU). Airflow obstruction and pulmonary hyperinflation were measured by forced expiratory volume in one second (FEV_1_) and residual volume (RV) according to ATS/ERS guidelines [14, 15]. Computational YACTA analysis (“yet another CT scan analyser”) [16] of high-resolution chest CT scans at BL and 90d-FU was performed to quantify the emphysema indexes of RUL + ML and RLL (EI_RUL+ML_ and EI_RLL_), e.g. the percentage of low attenuation areas < -950 Hounsfield units (HU), as well as the volumes of RUL, RUL + ML and RLL (Vol_RUL+ML_ and Vol_RLL_) at full inspiration. The emphysema volume of RUL + ML or RLL was then calculated as EI_RUL+ML_/100 × Vol_RUL+ML_ or EI_RLL_/100 × Vol_RLL_. Lung emphysema was classified as heterogeneous if there was a minimum of 10% points (pp) difference in destruction between RUL + ML and the ipsilateral non-targeted RLL., i.e. EI_RLL_ - EI_RUL+ML_ ≥ 10pp [11]. Fissure integrity was evaluated visually by an experienced thoracic radiologist. Perfusion scintigraphy was performed at BL [11]. Responder rates were evaluated based on the minimal clinical important difference (MCID) for FEV_1_ (increase ≥ 12%), RV (decrease ≥ 8.6%) and 6-MWD (increase ≥ 26 m) [17–22]. Percent change of outcome parameters was calculated from measurement results X_BL_ at baseline and _FU_ at follow-ups as: Percent change = (X_FU_ – X_BL_)/ X_BL_ × 100.

### Statistical analysis

Data were analysed with R 4.3.1 (R Core Team. R: A language and environment for statistical computing. R Foundation for Statistical Computing. *Vienna Austria*). Data were analysed for normality using Shapiro-Wilk test and reported as median [25–75th percentile] or mean ± standard deviation otherwise. Descriptive statistical analysis was performed using paired or unpaired Wilcoxon rank sum test, Student’s t-test or Pearson’s product-moment correlation as appropriate. Due to the nature of the retrospective analysis, the number of measurements (n) varied according to the availability of source data and *n* is reported individually. *P*-values less than 0.05 were considered statistically significant.

## Results

### Patient characteristics

From 12/2012 to 09/2023, a total of 28 patients with follow-ups were treated with combined RUL-ML valve implantation at Thoraxklinik Heidelberg, 26 patients sequentially in two separate bronchoscopies and 2 patients simultaneously in one procedure. Two patients received significant parts of their treatment externally and could not be included: RUL treatment for one patient and follow-up care for the other.

**Fig. 1:**
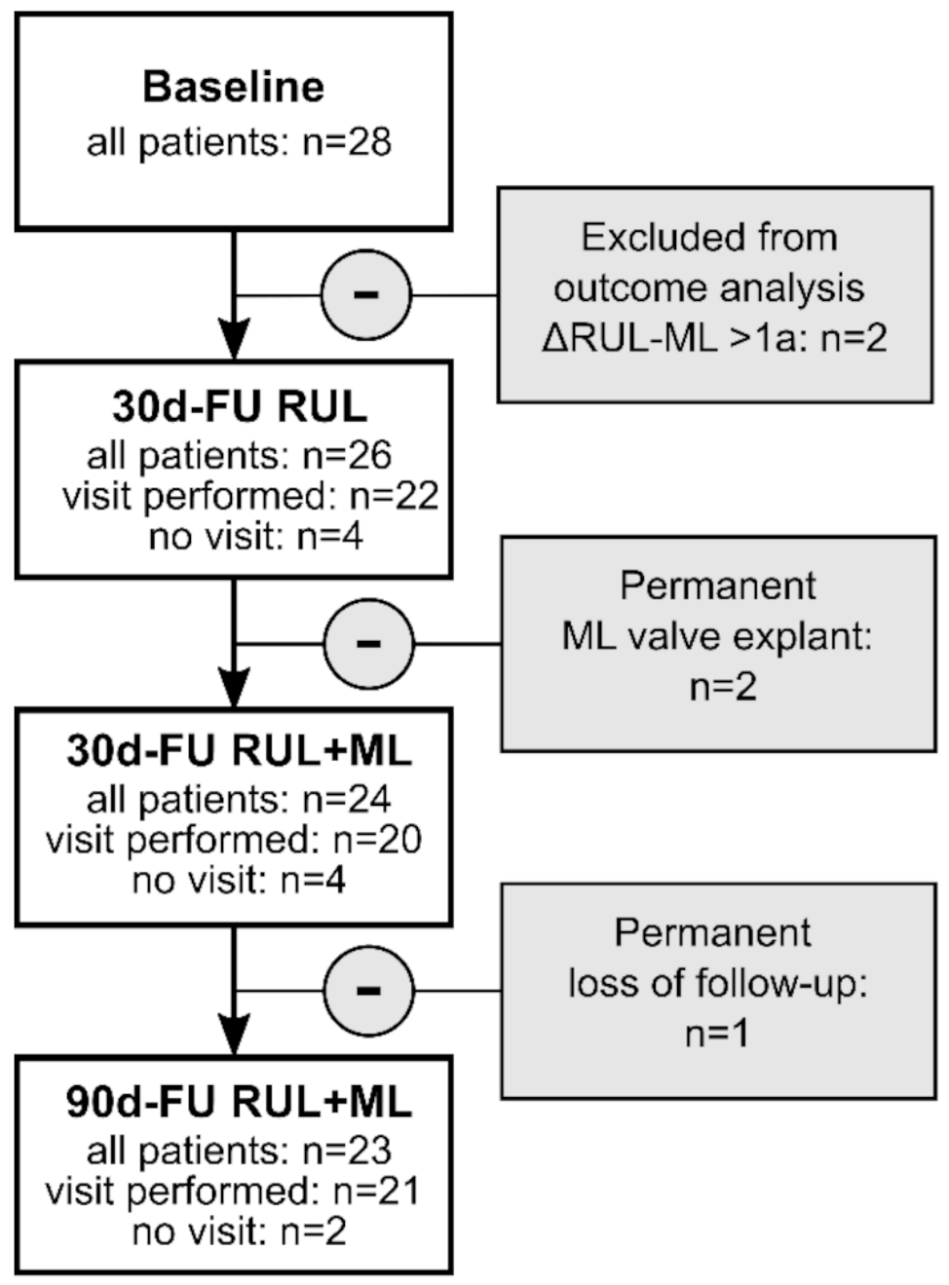
Patients included in the outcome analysis. 28 patients were included in the study. For two patients, the period between RUL and subsequent ML treatment exceeded 1 year and they were therefore excluded from the outcome analysis. Measurement data were available for 22 patients at 30 days after RUL treatment (30d-FU RUL), for 20 patients at 30 days after ML treatment (30d-FU RUL-ML), and for 21 patients at 90 days after ML treatment (90d-FU RUL + ML)

Baseline patient characteristics are summarized in Table 1: The included cohort fulfilled the criteria for ELVR [11] with severe obstructive ventilation disorder and massive pulmonary hyperinflation as well as reduced physical exercise capacity in the 6-MWD. RUL and ML represented an acceptable combined target as the right major fissure was intact (95.00 [90.00–95.00] %), the minor fissure was incomplete (70.00 [50.00–81.30] %) and both, RUL and ML, had increased emphysematous tissue. Perfusion was quantified at three lung heights independent of anatomical fissures and was significantly limited in the right upper lung area. 28.57% of patients had homogeneous and 71.43% heterogeneous emphysema. The majority of patients suffered from at least one COPD exacerbation in the preceding year. One patient was lost of follow-up before 90d-FU (Fig. 1). One patient had already been treated with valves in the RUL and ML before and the valves had been explanted 7 years before re-baseline. In 2 patients, sequential closure of the ML was performed as an individual healing attempt more than 1 year after the RUL treatment. These patients were analysed for safety but not for outcome.

**Table 1:** Baseline characteristics of study population and procedure information

**Age (years)**, Mean ± SD / Range	61.95 ± 6.87 / 46.35–78.75	n_total_=28
**Sex** males vs. females, n_subgroup_ (%)	18 vs.10 (64.28% vs. 35.71%)	n_total_=28
**FEV**_**1**_ **pred. (%)**, Mean ± SD / Range	28.46 ± 6.39 / 16.00–42.00	n_total_=28
**RV pred. (%)**, Mean ± SD / Range	261.60 ± 65.18 / 154.00–386.00	n_total_=28
**mMRC score (points)**, Median (IQR) / Range	3.00 [3.00–4.00] / 1.00–4.00	n_total_=21
**Minor fissure integrity (%)**, n_subgroup_ **(%)** **20%** **30%** **50%** **60%** **70%** **80%** **85%** **90%** **100%**	1 (3.57%) 2 (7.14%) 8 (28.57%) 2 (7.14%) 3 (10.71%) 5 (17.86%) 2 (7.14%) 3 (10.71%) 2 (7.14%)	n_total_=28
**Right major fissure integrity (%)**, n_subgroup_ **(%)** **> 95% − 100%** **95%** **93%** **90%** **85%** 80%* 75%*	10 (35.71%) 5 (17.86%) 2 (7.14%) 6 (21.43%) 3 (10.71%) 1 (3.57%) 1 (3.57%)	n_total_=28
**Perfusion right upper field (%)**, Mean ± SD / Range	6.15 ± 2.56 / 1.00–10.00	n_total_=26
**Perfusion right middle field (%)**, Mean ± SD / Range	20.73 ± 4.03 / 11.00–27.00	n_total_=26
**Perfusion right lower field (%)**, Mean ± SD / Range	17.88 ± 5.07 / 6.00–26.00	n_total_=26
**Emphysema index RUL & ML (%)**, Mean ± SD / Range	49.75 ± 12.36 / 12.00–69.00	n_total_=28
**Emphysema index RLL (%)**, Mean ± SD / Range	31.50 ± 14.03 / 4.00–55.00	n_total_=28
**Volume RUL & ML (L)**, Mean ± SD / Range	2.22 ± 0.66 / 1.42–4.52	n_total_=28
**Percentage Volume (RUL & ML)/RLL (%)**, Mean ± SD / Range	149.51 ± 56.01 / 89.56–332.53	n_total_= 28
**Volume RLL (L)**, Mean ± SD / Range	1.55 ± 0.33 / 0.92–2.15	n_total_=28
**Emphysema distribu**tion homogeneous vs. heterogeneous, n_subgroup_ (%)	8 vs. 20 (28.6% / 71.4%)	n_total_=28
**AECOPDs/ year**, n_subgroup_ **(%)** 0 1 2 ≥ 3	2 (8.0%) 13 (52.0%) 5 (20.0%) 5 (20.0%)	n_total_=25
**Valves RUL**, n_subgroup_ **(%)** Zephyr valves Spiration valves Zephyr and Spiration valves	24 (85.7%) 3 (10.7%) 1 (3.6%)	n_total_=28
**Valves ML**, n_subgroup_ **(%)** Zephyr valves	28 (100.0%)	n_total_=28

Definition of abbreviations: IQR: interquartile range 25–75th percentile. SD: standard deviation. n_subgroup_: number of patients in subgroup, n_total_: number of patients with available data. RLL: right lower lobe. RUL: right upper lobe. AECOPD: acute exacerbation of COPD

*Chartis® measurement performed in all cases, RUL CV-negative and treatment of middle lobe as second target lobe > 1 year after RUL treatment because of decreasing benefit

### Procedure details

The relationship between the performed Chartis^®^ measurements and fissure integrity is summarised in Table 2. In 9 of 11 patients (81.82%) with a ≤ 90% intact right major fissure, a Chartis^®^ measurement was performed. Five of these patients showed a clear absence of collateral ventilation in the RLL and one patient showed a sudden low flow phenomenon, which was interpreted as negative collateral ventilation (Table 2) [23]. The 2 patients who underwent an individual treatment attempt with the ML as second target lobe after more than one year were initially assessed as negative collateral ventilation for the RUL (Table 2). Another patient, who initially had negative RUL collateral ventilation despite a minor fissure integrity of 80%, underwent sequential ML treatment within 187 days after three RUL valve implantations due to lack of benefit (Table 2).Only 2 patients were treated simultaneously with valves in the ML and the RUL. In this small sub-cohort, no bronchoscopic valve control was required until 90 days after the procedure. In the majority of patients, physicians decided for a sequential approach, treating the RUL first and then the ML within 83.00 [49.00–132.80] days (range 0 to 784.0 days). Exclusively Zephyr valves were implanted in 85.71% of the RULs and in all treated MLs (Table 1). In 14.28% of RUL treatments, at least one Spiration valve was implanted (Table 1).

**Table 2:** Endoscopic measurement of collateral ventilation

		Right Major Fissure Integrity			
		> 95–100%	> 90–95%^§^	90%	< 90%
**Chartis**^**®**^ **performed**	*n*/*n*_subgroup_ (%)	2/10 (20.00%)	2/7 (28.57%)	5/6 (83.33%)	4/5 (80.00%)
RUL CV + *and* RLL CV-	*n*	1*	0	3	0
RLL CV-	*n*	0	0	1	1
RUL CV + *and* RLL low flow	*n*	1*^#^	2^#^	0	1^#^
RUL CV-	*n*	0	0	0	2^$^
RUL low flow	*n*	0	0	1°	0
**Chartis**^**®**^ **not performed**	*n*/*n*_subgroup_ (%)	8/10 (80.00%)	5/7 (71.43%)	1/6 (16.67%)	1/5 (20.00%)

Definition of abbreviations: CV+: positive collateral ventilation in Chartis^®^ measurement. CV-: absence of collateral ventilation in Chartis^®^ measurement. RLL: right lower lobe. RUL: right upper lobe. ML: middle lobe

*Integrity of minor fissure ≥ 90 to 95%

°Initially unsuccessful RUL treatment, most likely due to a minor fissure integrity of only 80%; after 3 RUL valve replacements decision for a sequential RUL-ML approach as an individual therapy concept

^$^Treatment of middle lobe > 1 year after RUL treatment as second target lobe because of decreasing benefit

^#^performed in 2014 to 2015

^§ 2^ patients 93%, 5 patients 95% major fissure integrity

In two of the sequentially treated patients, the valves in the RUL had to be replaced once or twice due to malfunction before the ML treatment could be continued. ML valves had to be explanted due to pneumothorax in 3 patients, of which 1 was reimplanted (Fig. 1). In two patients treated sequentially as an individual therapy attempt, the time between RUL and ML treatment was extended by 1 year. Because of the expected progression of COPD lung disease, these 2 patients were not included in the outcome evaluation (Fig. 1), but in the safety analysis.

### Outcome parameters

**Fig. 2:**
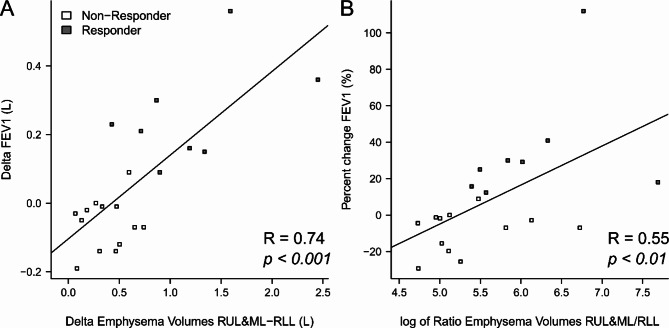
Association of emphysema volumes at baseline and response in FEV1 90 days after combined right upper lobe (RUL) and the middle lobe (ML) valve implantation. (**A**) Difference between FEV1 at 90d-FU and at baseline vs. the difference between the combined emphysema volume of ML and RUL and the emphysema volume of the RUL. (**B**) Percent change in FEV1 at 90d-FU from baseline vs. the ratio of the combined emphysema volume of ML and RUL to the emphysema volume of the RUL. Of note, emphysema quantification with YACTA analysis from baseline CTs correlated with response in FEV1 90 days after completion of treatment

At 90d-FU, there was no statistically significant increase of mean FEV_1_ and only a trend towards a reduction in RV in the overall cohort (Table 3). However, 8/21 patients (38.10%) reached the minimal clinically important difference (MCID) for FEV_1_ [17, 18]. The MCID for RV was achieved in 9 of 21 patients (42.86%) at 90d-FU. In the total cohort, we could not detect any effect on 6-MWD or mMRC (Table 3). Further analysis revealed that the absolute change in FEV1 at 90d-FU correlated strongly with the difference in emphysema volumes between the RUL + ML and the ipsilateral RLL at baseline (*R* = 0.74, *p* < 0.001, Fig. 2A). This association remained when we correlated the relative ratio of emphysema volumes with the percentage of change in FEV1 at 90d-FU to exclude any bias due to absolute lung volume (*R* = 0.55, *p* < 0.01, Fig. 2B). Further, the target lobe (RUL + ML) emphysema volume (*R* = 0.57, *p* < 0.01), total volume (*R* = 0.56, *p* < 0.01), but not the emphysema index (*R* = 0.37, *p* < 0.1), were associated with the absolute FEV1 change at 90d-FU.

**Table 3:** Outcome analysis of total cohort and patient subgroup with heterogenous emphysema

	Baseline	30d-FU RUL	30d-FU RUL & ML	90d-FU RUL & ML
**Total Cohort**				
Patients *N*	26	22	20	21
FEV_1_(L)	0.77 ± 0.23 *n* = 26	0.76 ± 0.22 *n* = 22	0.91 ± 0.33 *n* = 20, *p* = 0.07	0.87 ± 0.29 *n* = 21
RV (L)	5.38 ± 1.27 *n* = 26	5.66 ± 1.46 *n* = 22, *p* = 0.08	4.84 ± 1.13 *n* = 20, *p* = 0.08	4.94 ± 1.11 *n* = 21, *p* = 0.06
6-MWD (m)	282.10 ± 68.76 *n* = 24	288.20 ± 69.42 *n* = 19	323.70 ± 83.94 *n* = 16, *p* = 0.098	304.20 ± 97.87 *n* = 19
mMRC score (points)	3.00 [2.50–3.50] *n* = 19	3.00 [2.75–4.00] *n* = 16	3.00 [2.00–4.00] *n* = 15	3.00 [2.00–4.00] *n* = 15
**Subgroup with heterogenous emphysema**				
Patients *N*	19	16	15	15
FEV_1_(L)	0.77 ± 0.24 *n* = 19	0.76 ± 0.23 *n* = 16	0.94 ± 0.36 *n* = 15, *p* = 0.065	0.93 ± 0.31 *n* = 15, *p* < 0.05
RV (L)	5.43 ± 1.21 *n* = 19	5.92 ± 1.47 *n* = 16, *p* = 0.09	5.04 ± 1.16 *n* = 15	5.10 ± 1.16 *n* = 15
6-MWD (m)	284.47 ± 69.05 *n* = 17	297.64 ± 71.97 *n* = 14	338.92 ± 86.29 *n* = 12, *p* < 0.05	340.23 ± 66.37 *n* = 13, *p* < 0.05
mMRC score (points)	3.00 [2.50–3.50] *n* = 15	3.00 [2.50–4.00] *n* = 12	2.50 [1.75–3.25] *n* = 12	3.00 [1.50–3.50] *n* = 11
**Subgroup with fissure major integrity > 95% and CV- in Chartis** ^**®**^				
Patients *N*	15	12	9	12
FEV_1_(L)	0.78 ± 0.24 *n* = 15	0.80 ± 0.22 *n* = 12	1.046 ± 0.34 *n* = 9, *p* = 0.071	0.92 ± 0.29 *n* = 12
RV (L)	5.43 ± 1.22 *n* = 15	5.59 ± 1.39 *n* = 12	4.43 ± 0.83 *n* = 9, *p* < 0.05	4.68 ± 1.14 *n* = 12, *p* < 0.05
6-MWD (m)	272.30 ± 75.11 *n* = 14	284.00 ± 75.86 *n* = 8	349.40 ± 89.04 *n* = 7	285.1 ± 133.23 *n* = 12

Definition of abbreviations: RUL: right upper lobe. ML: middle lobe. CV-: absence of collateral ventilation in Chartis^®^ measurement

**Fig. 3:**
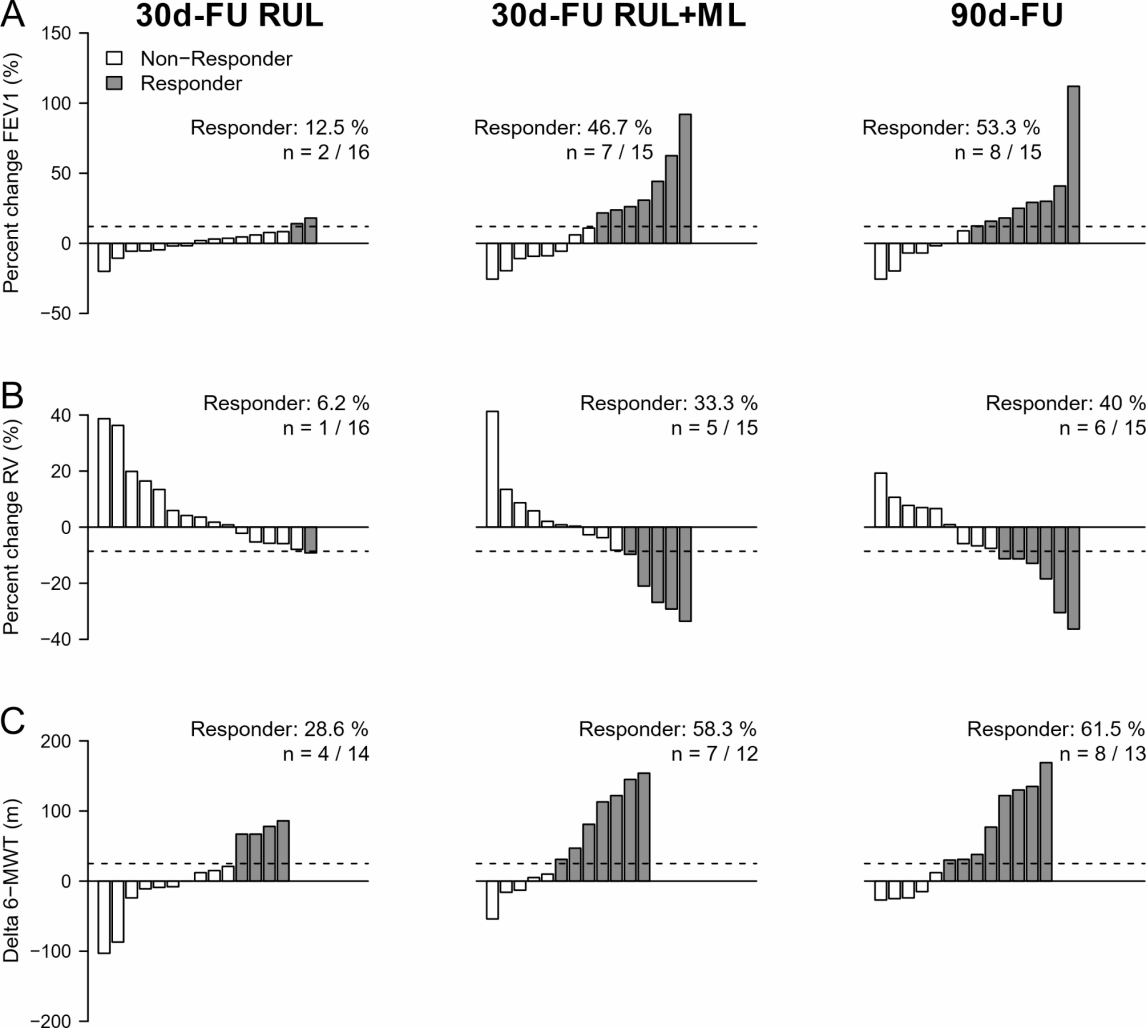
Waterfall plots of the subjects with heterogenous emphysema at 30 days after treatment of the right upper lobe (30d-FU RUL) and at 30 or 90 days after completion of combined treatment of the right upper lobe and middle lobe (30d-FU RUL + ML and 90d-FU) compared to baseline for percent change in FEV1 (**A**), percent change of RV (**B**) and absolute change of 6-minute walking test (**C**). The minimal clinical important distance (MCID) is shown as a dashed line for FEV1 (≥ 12% increase, **A**), RV (≥ 8.6% decrease, **B**) and 6-minute walking test (≥ 26 m increase, **C**). Non-responders (below MCID level) are displayed in white and responders in grey

Based on this association with heterogeneity, we repeated the response analysis in the subgroup with heterogeneous emphysema (*N* = 19 at baseline), which showed a significant FEV1 increase (Table 2) of 116.00 mL ± 195.77 mL (*n* = 15) at 90 days after completion of combined RUL-ML treatment. Responder rates were 53.30% (*n* = 8/15) for FEV1 and 40.00% (*n* = 6/15) for RV at 90d-FU (Fig. 3). Also exercise capacity improved significantly in the subgroup with heterogeneous emphysema at 30 and 90 days after RUL-ML treatment by 52.08 ± 69.44 m and 50.23 ± 69.10 m (Table 2). The MCID [19, 20] was exceeded in 7 of 12 patients (58.33%) and in 8 of 13 patients (61.54%) accordingly (Fig. 3). In some cases, although the visit had taken place, no values were available for the 6-MWD and the reasons for this could not be determined retrospectively (Table 3). As shown in Table 4, the significant improvements in FEV_1_ and 6-MWD did not show large differences from the expected range of published data. In contrast to the patients with heterogenous emphysema, there were no significant effects in patients with homogeneous emphysema (*n* = 7). We also studied the subgroup which met the current criteria for negative collateral ventilation (major fissure integrity > 95%, clear negative Chartis^®^ signal in the RLL). Although the subgroup was small (*n* = 15), we noted a significant decrease in RV. In the other parameters, no effects could be shown.

**Table 4:** Comparison of outcome parameters with randomized controlled trials [5, 7, 8, 10, 27–29, 31]

Trial	Measure	Month	Emphysema distribution	ΔFEV_1_ (ml)	Resp. rate FEV_1_ (%)	ΔRV (ml)	Δ6MWT (m)
VENT (2010)	Mean (95% CI)	6	Not specified	34.5 (10.8–58.3)	-	-	9.3 (-0.5–19.1)
STELVIO (2015)	Mean (95% CI)	6	Homogenous and heterogenous	161 (80–242)	59 ^§^	-	60 (35–85)
BeLieVeR-HiFi (2015)	Median [IQR]	3	heterogenous	60 [20–380]	39 *	-260 [-1070–160]	25 [7–64]
IMPACT (2016)	Mean ± SD	3	homogenous	100 ± 180	39.5 ^#^	-420 ± 900	22.6 ± 66.6
TRANSFORM (2017)	Mean ± SD	3 6	heterogenous	150 ± 200 140 ± 240	- 56.3 ^#^	- -660 ± 1040	37.2 ± 65.1 36.2 ± 76.9
LIBERATE (2018)	Mean ± SD	12	heterogenous	104 ± 200	56.4 ^#^	-490 ± 830	12.98 ± 81.5
EMPROVE (2019)	Mean ± SD	6 12	heterogenous	99 ± 154 67 ± 167	43.4 ^#^ 41.9 ^#^	-402 ± 849 -	-4.4 ± 76.7 -
REACH (2019)	Mean ± SD	3 6	heterogenous	104 ± 178 91 ± 156	48 ^#^ 41 ^#^	-520 ± 1430 − 420 ± 1840	27.17 ± 72.0 20.82 ± 86.7
**RUL-ML STUDY**							
Total cohort	Mean ± SD	3	Not specified	61.9 ± 189.4 (n.s.)	38.1 ^#^	-400.9 ± 916.0 (n.s.)	12.4 ± 104.0 (n.s.)
Heterogenous subgroup	Mean ± SD	3	heterogenous	116.0 ± 195.8	53.3 ^#^	-387.3 ± 853.4 (n.s.)	50.2 ± 69.1

### Adverse effects

Pneumothorax requiring chest tube draining occurred in 6 cases, one after RUL and 5 after ML treatment (Table 5). The pneumothorax, which occurred after RUL treatment, was recorded in one of the patients who was initially treated only in the RUL due to negative RUL collateral ventilation, and after > 1 year was treated in the middle lobe as a second target lobe. In one of the cases occurring after ML treatment, ML valves had to be explanted and later reimplanted. In two patients, post-interventional pneumothorax after ML therapy led ultimately to permanent explantation of the ML valves without reimplantation (Fig. 1; Table 5). In one patient, a mild pneumothorax after ML therapy did not require any further intervention. (Table 5). As expected based on COPD exacerbation history (Table 1), 50% (*n* = 14/28) of patients experienced at least one mild to moderate respiratory infection and/or COPD exacerbation during the course of the study. Severe pneumonias and/or COPD exacerbations with need of hospitalization and/or intravenous therapy occurred in 32.1% of patients (*n* = 9/28), but none of these occurred within 7 days of a procedure. In addition, 14.3% (4/28) of subjects experienced mild to moderate haemoptysis.

**Table 5:** Respiratory adverse events until 90 days after completion of RUL-ML treatment

	Complete Study period		RUL to ML treatment		ML treatment to 90d-FU*	
	Events *N*	Patients *n* (%)	Events *N*	Patients *n* (%)	Events *N*	Patients *n* (%)
**Severe events**						
Mortality	0	0 (0%)	0	0 (0%)	0	0 (0%)
Severe haemoptysis	0	0 (0%)	0	0 (0%)	0	0 (0%)
Pneumothorax with thoracic draining	6	5 (17.9%)	1	1 (3.6%)	5	4 (14.3%)
Severe pneumonia	2	2 (7.1%)	2	2 (7.1%)	0	0
Severe AECOPD	7	5 (17.9%)	4	4 (14.3%)	3	2 (7.1%)
Severe pneumonia and AECOPD	3	2 (7.1%)	2	2 (7.1%)	1	1 (3.6%)
**Mild to moderate events**						
Self-limiting haemoptysis	4	4 (14.3%)	0	0 (0%)	4	4 (14.3%)
Pneumothorax without intervention	1	1 (3.6%)	0	0 (0%)	1	1 (3.6%)
Mild to moderate respiratory infection	6	5 (17.9%)	5	5 (17.9%)	1	1 (3.6%)
Mild to moderate AECOPD	10	7 (25.0%)	7	6 (21.4%)	3	2 (7.1%)
Mild to moderate respiratory infection and AECOPD	9	7 (25.0%)	6	4 (14.3%)	3	3 (10.7%)
**Re-bronchoscopies**						
Re-bronchoscopy indicated	18	15 (53.6%)	7	6 (21.4%)	11	11 (39.3%)
Refused by patient	2	2 (7.1%)	0	0 (0%)	2	2 (7.1%)
Valves fully functional	5	4 (14.3%)	3	3 (10.7%)	2	2 (7.1%)
Valves secretion-sealed	2	2 (7.1%)	0	0 (0%)	2	2 (7.1%)
Valve replacement (same procedure)						
Due to dysfunction	2	2 (7.1%)	2	2 (7.1%)	0	0 (0%)
Valve interval reimplantation						
Due to pneumothorax	1	1 (3.6%)	0	0 (0%)	1	1 (3.6%)
Due to dysfunction	3	2 (7.1%)	2	1 (3.6%)	1	1 (3.6%)
Permanent valve explantation						
Due to pneumothorax	2	2 (7.1%)	0	0 (0%)	2	2 (7.1%)
Due to limited response	1	1 (3.6%)	0	0 (0%)	1	1 (3.6%)
**No re-bronchoscopy performed**	NA	13 (46.4%)	NA	NA	NA	NA

Definition of abbreviations: RUL: right upper lobe. ML: middle lobe. AECOPD: acute exacerbation of COPD

* If the decision to re-bronchoscopy was made up to 90d-FU, the outcome of re-bronchoscopy was analysed

Re-bronchoscopy to check valve function and position was indicated until the 90d-FU and subsequently performed at least once in 11 patients (39.3%, 13 events). In further 2 patients, this procedure was offered but not performed for personal patient reasons (Table 5). These re-bronchoscopies showed that the valves were fully functional in 5 cases. Valves were secretion-sealed and needed to be cleaned in 2 cases. Permanent valve explantation due to limited response was performed in 1 case (Table 5).

Due to lack of benefit and endoscopically seen dislocation, valve replacement was necessary in 2 patients and valve explantation with later reimplantation in a further 2 patients (3 events, Table 5). 3 of these 4 patients with proven valve dysfunction underwent re-bronchoscopies after RUL treatment: (i) In the first patient, a Zephyr valve in RB2 migrated. (ii) In the second patient, a Spiration valve that was initially implanted in the right segmental bronchus (RB) 1 and RB2 no longer fully covered RB1. (iii) The third patient had a very steeply sloping RB1 and was treated with Zephyr valves in RB1a, RB1b, RB2 and RB3. On re-bronchoscopy, the valves in RB1a, RB1b and RB2 were dislocated and explanted. 2 Spiration valves were subsequently implanted in RB1a and RB1b and a Zephyr valve in RB2. Due to lack of benefit, re-bronchoscopy was performed again, valves were dysfunctional due to mucus obstruction and completely removed. In two further procedures, RUL and subsequent ML treatment finally could be completed.

After ML-treatment, 1 of the 4 patients with valve dysfunction underwent Zephyr valve explantation and re-implantation due to granulation tissue. As far as comparable, this real-world cohort experienced more adverse events than the selected RCT patients (Table 6).

**Table 6:** Proportion of patients with adverse events in randomized controlled trials and current analysis [5, 7, 8, 10, 27–29, 31]

Trial	FU-Time (months)	Mortality	Pneumo-thorax	Valve migra-tion	Pneumonia	Severe AECOPD	AECOPD (total)	Mild to moderate AECOPD
VENT (2010)	6	0.9%	4.2%	4.7%	3.2%	7.9%	-	1.4%
STELVIO (2015)	6	2.9%	17.6%	5.9%	5.9%	11.8	-	44.1%
BeLieVeR-HiFi (2015)	3	8%	8.0%	16.0%	8.0	-	64.0%	-
IMPACT (2016)	3	0%	25.6%	4.6%	0%	16.3%	-	-
TRANSFORM (2017)	< 1 1–6	1.5% 0%	20% 3.1%	- 1.5%	4.6% 4.6%	-	4.6% 4.6%	-
LIBERATE (2018)	< 1.5 1.5–12	3.1% 0.8%	26.6% 6.6%	3.9%	0.8% 5.7	-	7.8% 23.0%	-
EMPROVE (2019)	< 6 6–12	0% (related) 1.0% (related) 5.3% (total)	14.2% 1.0%	0%	8.9% 8.8	-	16.8% 13.6%	-
REACH (2019)	6	0%	7.6%	0%	1.5	-	7.6% (related) 12.1% (unrelated)	-
RUL-ML STUDY	3	0%	21.4%	17.9%*	7.1%^#^	25.0%^#^	46.4%^#^	42.9%^#^

Definition of abbreviations: FU: follow-up, AECOPD: acute exacerbation of COPD, RUL: right upper lobe, ML: middle lobe

*Including patients with elective valve control for non-response indicated at 90d-FU

^#^Cases of severe or moderate COPD exacerbation with antibiotic treatment were in this table assigned to the category severe AECOPD or moderate AECOPD, respectively

**FIGURES AND FIGURE LEGENDS**

## Discussion

In this study, we systematically analysed the effectiveness and safety of combined valve treatment of the right upper lobe and middle lobes in a retrospective real-world emphysema cohort. Previous work shows that sequential treatment of the middle lobe is already used in clinical practice [24, 25]. However, to our knowledge, this is the first systematic analysis with the main focus on this approach, which increases the proportion of patients who can be offered valve therapy despite an incomplete minor fissure. The main findings of our study were that RUL-ML treatment can improve lung function in patients with right-sided heterogenous emphysema, may have beneficial effects on exercise capacity and is associated with an acceptable risk profile in patients with indication for endoscopic lung volume reduction.

The ideal target lobe for valve implantation is characterized by a high emphysema proportion as well as air-trapping in high-resolution CT scans with reduced perfusion in lung scintigraphy and complete adjacent fissures, indicating the absence of collateral ventilation [3, 11–13, 26]. Unfortunately, in clinical routine all of these ideal conditions are often not met and a substantial proportion of patients with an indication for ELVR cannot be treated with neither Zephyr or Spiration valves. In order to offer this well-established and, above all, relatively safe and reversible procedure to a larger patient population [7–10, 27–31], at Thoraxklinik Heidelberg combined valve implantation in both, the RUL and ML, has been performed in a cohort of selected patients with a complete major, an incomplete minor fissure and radiological evidence of a dysfunctional RUL and ML (Table 1). We therefore conducted a retrospective analysis to investigate the outcome and the safety of this strategy in patients treated 2012 to 2023. In the majority of cases, a sequential approach was chosen, with the RUL treated first and the ML treated after an interval of 112.00 [51.00–157.50] days. This approach was preferred to minimise the risk of post-interventional complications, predominately pneumothorax. However, in two individual cases, a simultaneous strategy was also successful. In 2 patients, the interval between RUL and ML treatment was longer than 1 year, and the follow-up period after baseline would be significantly exceeded. These patients were therefore not included in the outcome, but in the safety analysis.

From a mechanistic point of view, the greatest therapeutic success after combined lung volume reduction of RUL and ML would be expected if the RLL had a relatively large proportion of intact lung tissue to occupy the right hemithorax after successful valve implantation [26]. The importance of emphysema heterogeneity for treatment response after endoscopic lung volume reduction has been repeatedly confirmed and has therefore been a selection criterion in most randomised controlled valve therapy trials [5–7, 10, 26–28, 32–35]. Also, in our study, the majority of patients selected for combined RUL-ML valve implantation (71.4%) showed a heterogeneous emphysema distribution. The post-interventional increase in FEV1 correlated with the absolute and relative relationships between the emphysema volumes of RUL plus ML and RLL in the pre-interventional baseline CT scans: The greater the amount of healthy RLL tissue relative to the target lobes in quantitative CTs before treatment, the greater was the FEV1 response after treatment (Fig. 2). This is in line with the results of a recent study showing correlations between emphysema heterogeneity and outcome parameters after endobronchial valve treatment [34]. In the sub-cohort with heterogeneous emphysema, we then observed significant increases in FEV1 and 6-MWD at 90 days after completion of RUL-ML valve treatment, although the sub-cohort was small. These results were similar to findings from RCTs on Zephyr [7, 8, 28, 29] or Spiration valves [31, 35].

In rare cases, the middle lobe may even be considered the only target lobe. This was recently further evaluated by Klooster et al. [36], who performed a retrospective study of 15 patients treated with valves exclusively in the middle lobe. In this cohort, the median emphysema proportion (<-950 HU) of the middle lobe was 58% with a medium volume of 908 mL. After valve implantation, a significant target lobar volume reduction was observed in 86% of treated patients and responder rates at six months were 47% for FEV1, 50% for RV, and 64% for 6-MWD [36]. Our analysis yielded results in a comparable range, highlighting the potential of the middle lobe as a target for an ELVR, either alone or in combination.

As expected in presence of collateral ventilation, there was no significant response to RUL treatment alone, either in the overall population or in the heterogeneous emphysema subgroup. Chartis^®^ measurement is suggested in case of a gap ≥ 5–20% in the fissure adjacent to the target lung lobe [12]. The Chartis^®^ signal may be unambiguous, but interpretation may also be more difficult if the flow is interrupted immediately. In the studied cohort, both of these scenarios occurred and were classified as negative collateral ventilation. However, our retrospective analysis over a 10-year period in a small cohort does not allow a specific evaluation of the association between the Chartis^®^ signal structure and response to therapy. Although the subgroup with fissure major integrity > 95% and CV- in Chartis^®^ was small, we could show significant post-interventional reductions in RV. A significant proportion of patients with fissures = 95% did not undergo Chartis^®^ measurements. Based on the retrospective character of the manuscript, we have noted, that Chartis^®^ measurements were not performed for right major fissures with = 95% integrity in the older, first years. The bronchoscopist oriented himself according to the visual CT fissure analysis performed by the experienced radiologist and fissures with 95% integrity were in clinical practice considered as complete without being confirmed with a Chartis^®^ measurement.

Consistent with the lack of benefit, only one case of pneumothorax requiring intervention occurred directly after RUL treatment. In this patient, however, the sequential ML implantation was performed after more than one year as a second target lobe in an individual therapy attempt and was not intended initially. In contrast, 5 pneumothoraces occurred after completion of combined RUL-ML treatment, leading to permanent valve explantation in 2 cases and to valve explantation and later reimplantation in 1 case. As the clinical decision for endoscopic valve control is usually made at 90d-FU, we also analysed the results of these procedures even if they were performed after 90d-FU. Irrespective of the RUL-ML approach, the expected valve complications such as migration or dysfunction occurred in 17.9% of patients [5–8, 27, 28]. Further, we recorded severe COPD exacerbations with (3.6%) and without pneumonia (7.1%) in 10.7% of patients within 90 days after completion of combined RUL-ML valve therapy. From Baseline to RUL treatment, these numbers were higher, according to longer observational periods, in two cases even longer than 1 year. This was not surprising, since lower respiratory tract infections and COPD exacerbations are a known complication and occur in up to 20% of patients within 3 month after valve treatment [3]. In our study, 93% of the patients underwent at least 2 interventions and the majority of patients were already suffering from recurrent COPD exacerbations before the onset of interventions (Table 1). Therefore, the patient history and the prolonged observational period might likely explain the high rate of respiratory events in our cohort. Conversely to this short-term observation, a current study had shown that endoscopic valve therapy can even lead to a reduction in the frequency of exacerbations in the long-term [24].

Summarising, the results show that combined valve therapy of the RUL and ML can be a justified therapeutic approach. The indication ideally may be a severely damaged RUL and ML, a complete right major fissure and an incomplete minor fissure, negative collateral ventilation of the RLL and positive collateral ventilation of the RUL, heterogeneous emphysema distribution as well as reduced perfusion in the affected areas. In some cases, it may be technically impossible to determine collateral ventilation invasively, which requires a careful interdisciplinary risk-benefit assessment.

Our study has some limitations. Firstly, because combined valve implantation in the RUL and ML is performed less frequently than treatment of a single lobe, the number of patients is small despite the long observation period. The analysis could possibly be underpowered and to obtain a more valid statement, an evaluation of a larger multicentre cohort would be useful. On the other hand, our data were sufficient to show significant effects in patients with heterogeneous emphysema. Secondly, this is a retrospective study and the data cannot reach the quality of a prospective evaluation. However, the clinical management of patients undergoing airway valve implantation is standardised, with defined follow-up schedules and specific protocols for the management of complications at the Thoraxklinik Heidelberg. Therefore, we believe that the data sets of the patients in this study are likely to be consistent. Furthermore, as one caveat of this retrospective setting, only available data could be used for the comparison versus baseline. Therefore, it was not possible to determine whether patients with incomplete follow-up data might have responded less favourably. To minimise bias, we used paired statistical tests. Another limitation might be the fact that, in clinical practice, patient benefit is the primary intention and the individual is considered holistically. Therefore, the inclusion criteria of this real-world study may deviate from the usual eligibility criteria in RCTs on ELVR [3]. Finally, CT fissure analysis does not provide absolute certainty of the absence of collateral ventilation. This was especially true in the older days when the precision of quantitative CT was lacking. This could have led in our real life study, to CV + patients possibly been treated, causing the RUL-ML strategy to appear less efficient. However, in clinical practice, the judgement of an experienced radiologist is currently still an essential part of the baseline evaluation for ELVR strategies and cannot be fully replaced by automated analysis.

## Conclusions

In conclusion, the current data show that combined implantation of valves in the RUL and ML might be an effective and safe treatment strategy for endoscopic lung volume reduction in patients with severe heterogenous emphysema with incomplete minor but complete right major fissures.

## Data Availability

All data generated or analyzed during this study are included in this article. Further enquiries can be directed to the corresponding authors.

## Abbreviations

30d-FU RUL: Follow-up visit 30 days after treatment of the right upper lobe
30d-FU RUL + ML: Follow-up visit 30 days after treatment of the middle lobe
6-MWD: Six-minute walking distance
90d-FU: Follow-up visit 90 days after treatment of the middle lobe
BL: Baseline
COPD: Chronic obstructive pulmonary disease
CT: Computed tomography
CV-: Absence of collateral ventilation
CV+: Presence of collateral ventilation
EI_RUL+ML_: Emphysema index of the right upper and middle lobe
EI_RLL_: Emphysema index of the right lower lobe
ELVR: Endoscopic lung volume reduction
FEV1: Forced expiratory volume in 1 s
HU: Hounsfield unit
IQR: Interquartile range 25–75th percentile
LLL: Left lower lobe
LUL: Left upper lobe
MCID: Minimal clinical important difference
mMRC: Modified medical research council score
ML: Middle lobe
pp: Percentage points
RB: Right segmental bronchus
RCT: Randomized controlled trial
RLL: Right lower lobe
RUL: Right upper lobe
RV: Residual volume
Vol_RUL+ML_: Volume of the right upper and middle lobe
Vol_RLL_: Volume of the right lower lobe
YACTA: Yet another CT scan analyser
